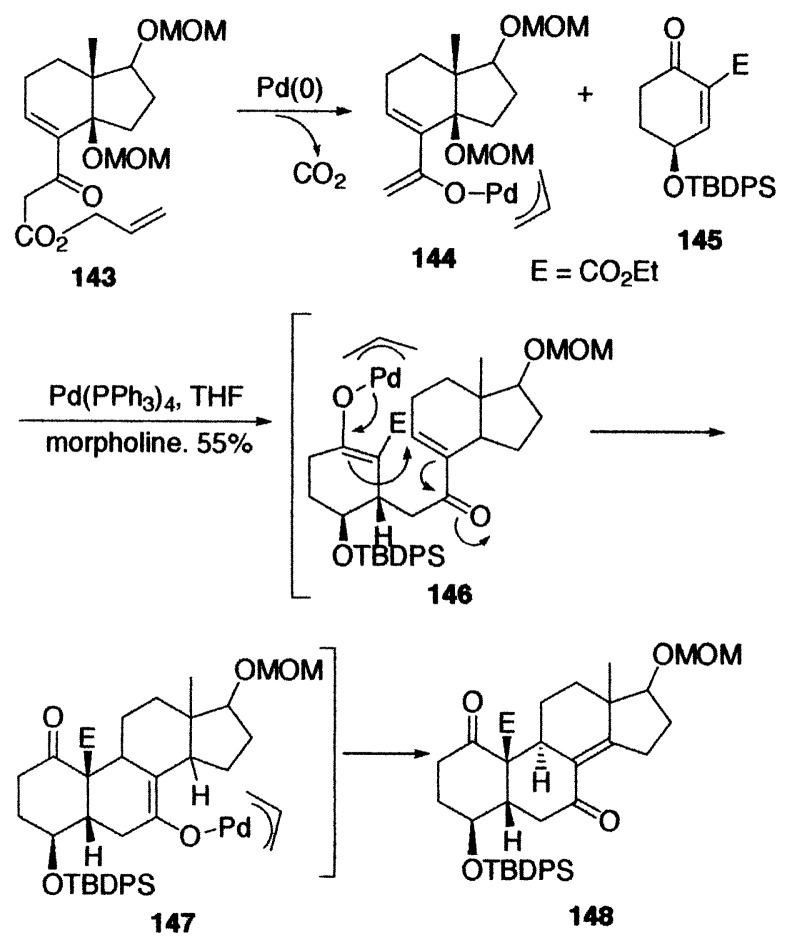# Development of ***β*** -keto ester and malonate chemistry: Palladium-catalyzed new reactions of their allylic esters

**Published:** 2004-08-01

**Authors:** Jiro Tsuji

**Affiliations:** Professor Emeritus, Tokyo Institute of Technology

**Keywords:** Carroll rearrangement, palladium enolates, palladium-catalyzed aldol condensation, palladium- catalyzed Michael addition

## Abstract

During extensive studies on ***π***-allylpalladium chemistry, we have developed classical ***β***-keto ester and malonate chemistry to a new generation by discovering a variety of palladium-catalyzed reactions of their allylic esters. Palladium enolates are generated from allyl ***β***-keto esters after decarboxylation and undergo the following transformations; a) reductive elimination to provide ***α***-allyl ketones, b) elimination of ***β***-hydrogen to give ***α***, ***β***-unsaturated ketones, c) formation of ***α***-methylene ketones, d) hydrogenolysis to give ketones, e) aldol condensation, and f) Michael addition. Allyl malonates and cyanoacetes undergo similar reactions. Results of these studies, including several applications carried out by other researchers are summarized.

## Introduction

Palladium is now regarded as the most versatile metal among a number of transition metals used for organic synthesis.1) We started research on organopalladium chemistry in early 1960s, and discovered carbon-carbon bond formation by using palladium complexes for the first time. Since then we have carried out extensive studies on organopalladium. One topic of these studies is palladium-catalyzed reaction of allylic compounds *via ****π***-allylpalladium complexes. Among a number of new catalytic reactions discovered in this area, synthetically useful catalytic reactions of allyl ***β***- keto carboxylates and malonates *via* palladium enolates are summarized in this review.

## The first palladium-mediated carbon-carbon bond formation

At first we investigated the possibility of carbon-carbon bond formation by using palladium complexes. When we treated the stable PdCl_2_ complex of cyclooctadiene (COD) **1** with diethyl malonate in ether in the presence of sodium carbonate at room temperature, a facile carbopalladation occurred to give the new stable complex **2**. This reaction is the first example of carbopalladation of an alkene in palladium chemistry, resulting in the carbon-carbon bond formation. The palladium-carbon ***σ***-bond in the complex **2** is stabilized by coordination of ***π***-olefin bond. By the treatment of the complex **2** with a base, malonate anion was generated and attacked the palladium-carbon bond, affording the bicyclo[6.1.0]nonane **3**. The reaction of the complex **2** with diethyl malonate gave rise to the bicyclo[3.3.0]octane **4** by transannulation.2)

**Figure:**
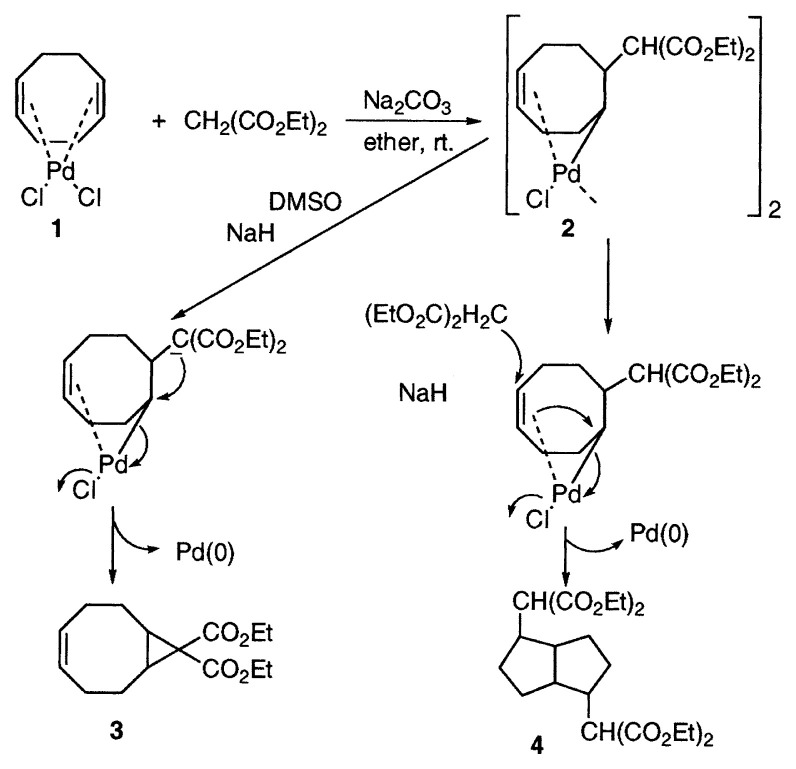


Then we discovered a nucleophilic attack of a carbanion to ***π***-allylpalladium chloride (**5**).3) The reaction of **5** with the carbanion generated by the treatment of diethyl malonate with NaH proceeded in DMSO, and diethyl allylmalonate (**6**) was obtained as expected with precipitation of palladium metal. Furthermore the reaction of ***π***-allylpalladium chloride (**5**) with the enamine **7** of cyclohexanone, which is regarded as a pseudo-carbanion, afforded 2-allylcyclohexanone after hydrolysis of the reaction product. These reactions constitute the basis of ***π***-allylpalladium chemistry. Then catalytic allylation of nucleophiles including carbon nucleophiles with allyl acetate and allyl phenyl ether to afford **8** was reported by two groups in 1970.4) Discovery of these reactions marked the birth of ***π***-allyl-palladium chemistry which has made steady and remarkable progress in the last thirty years.

**Figure:**
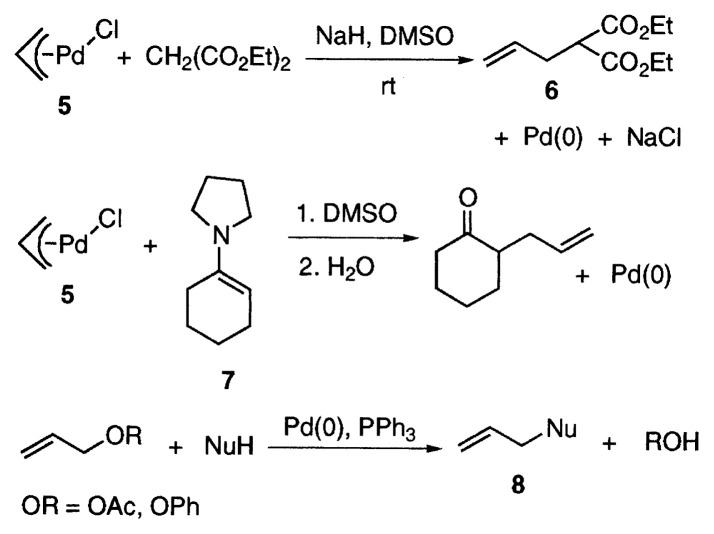


The facile reactions of PdCl_2_ complex of COD (**1**) and ***π***-allylpalladium chloride (**5**) with the carbon nucleophiles are significant in history of organometallic chemistry by the following reason. It is well-established that organometallic compounds known at that time, typically allylmagnesium halide, are nucleophilic, and react with carbonyl groups. At the same time Mg(II) is generated, showing that Grignard reaction involves the oxidation of Mg(0) to Mg(II). Thus the Grignard reaction is intrinsically stoichiometric, because *in situ* reduction of Mg(II) to Mg(0) is practically impossible. On the other hand, we have shown that ***π***-allylpalladium chloride (**5**) is electrophilic, offering a new concept in organometallic chemistry. The reaction of the palladium complexes with nucleophiles accompanies the reduction of Pd(II) to Pd(0). Formation of Pd(0) suggests the possibility of a catalytic reaction. The generation of Pd(0) after the reactions is the most characteristic feature of palladium complexes.

**Figure:**
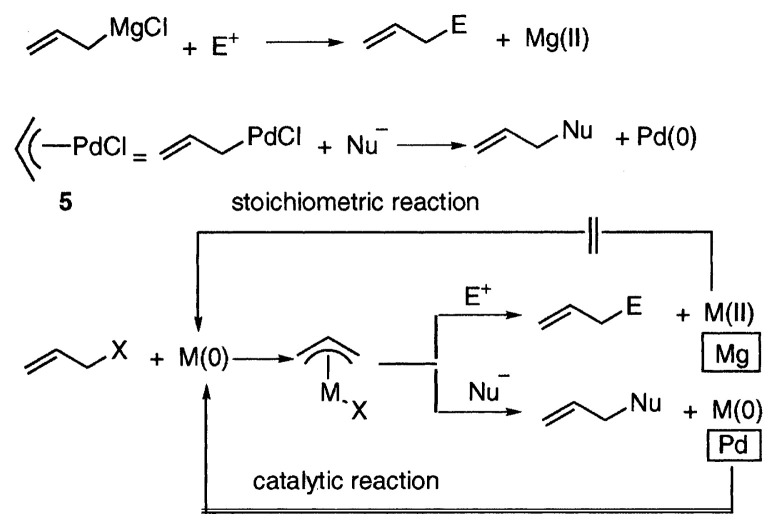


## Reactions of allyl ***β***-keto carboxylates and related compounds.5)

Acetoacetates and malonates are important compounds in organic chemistry. They are extensively used in organic synthesis for the preparation of a variety of ***β***-alkylated ketones **9**, esters **10**, and carboxylic acids **11** *via* alkylation, hydrolysis, and decarboxylation. We expected that acetoacetate (***β***-keto esters in general) and malonate chemistry can be expanded further by introduction of palladium-catalyzed reactions of their allylic esters. We found that ***π***-allylpalladium enolate **13** and ***α***-(***π***-allylpallada)ketones **14** are generated after facile decarboxylation by the treatment of allyl ***β***-keto carboxylates **12** with palladium catalyst, and undergo several transformations. Thus we expanded the usefulness of ***β***-keto esters based on the palladium-catalyzed reactions of their allylic ester, offering new synthetic methodologies which are not attainable by conventional methods. In addition to ***β***-keto esters, it was confirmed that allyl acetates **15** which have electron-withdrawing groups (EWG) such as alkoxycarbonyl, cyano, nitro, and sulfonyl groups at ***α***-carbon undergo similar transformations *via* **16**. In this review, palladium-catalyzed reactions of allyl ***β***-keto carboxylates **12** and allyl acetates **15** bearing EWG *via* the intermediates **13** and **16** are summarized.

In order to give a general view, six types of palladium-catalyzed reactions of allyl ***β***-keto carboxylates are summarized in the following scheme, citing allyl cyclohexanonecarboxylate **17** as a model compound. Allyl ***β***-keto carboxylate **17** undergoes palladium-catalyzed oxidative addition, followed by facile decarboxylation to form ***π***-allylpalladium enolate **18** and ***α***-(***π***-allylpallada) ketone **19**. Also **18** and **19** are generated from enol carbonates **20** of the corresponding ketones. When these reactions were discovered, almost nothing was known about palladium enolates, and we started to explore the chemistry of palladium enolates. The palladium enolates, generated in this way, undergo reductive elimination, ***β***-hydrogen elimination, and other transformations as expected. As summarized in the scheme, six transformations to afford the respective products **21**–**27** occur under different conditions and depending on the substituents R’s.5) In addition to allyl ***β***-keto carboxylates, other allyl acetates bearing electron-withdrawing groups at ***α***-carbon such as allyl malonates, cyanoacetates, and nitroacetates undergo similar transformations. Each transformation is explained in the followings.

**Figure:**
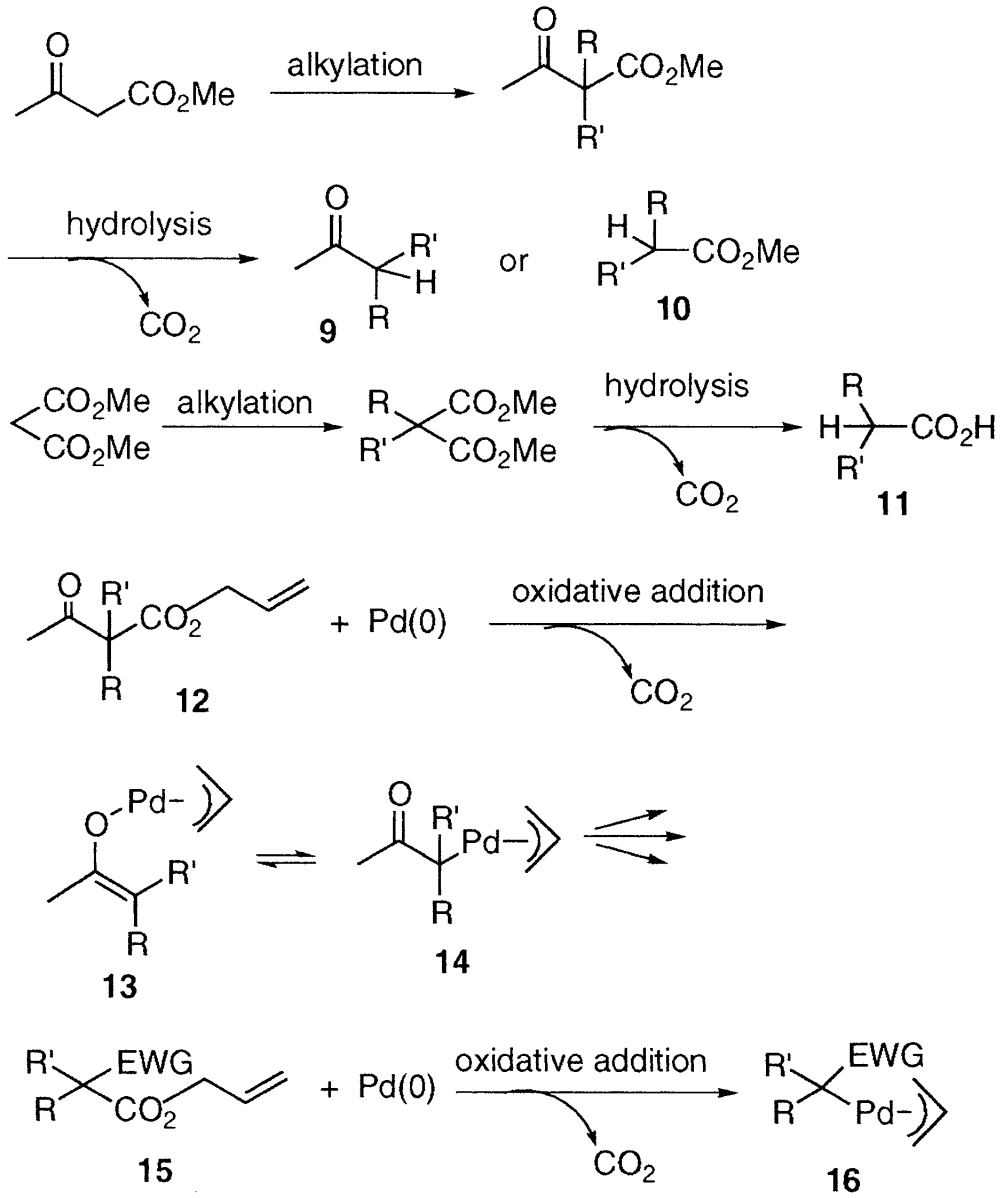


## Allylation by reductive elimination

The first reaction is decarboxylation-allylation of allyl ***β***-keto carboxylates **17** or acetoacetate **28** to afford ***α***-allyl ketones **21** by reductive elimination of **18**, or ***γ***, ***δ***-unsaturated methyl ketones **29**. Formation of allyl ketones **21** and **29** from allyl ***β***-keto esters **17** and **28** is known as the Carroll rearrangement, which is carried out at temperatures as high as 200 ˚C. The rearrangement is useful for the synthesis of terpenoids. We found that the palladium-catalyzed Carroll rearrangement proceeds smoothly under mild conditions.6)–8) Mechanism of the thermal rearrangement is explained by [3.3]sigmatropic rearrangement and palladium-catalyzed reaction proceeds *via ****π***-allylpalladium enolate formation. Difference in mechanisms can be shown by the reaction of allyl ***α***, ***α***-dimethyl acetoacetate (**30**). No thermal [3.3]sigmatropic rearrangement occurs because there is no possibility of enolization. On the other hand, the palladium-catalyzed rearrangement proceeds smoothly under mild conditions in THF to afford the ***α***-allyl ketone **31** regioselectively. Palladium-catalyzed reaction of geranyl acetoacetate (**32**) in THF afforded geranylacetone (**34**) selectively. On the other hand, a mixture of **34** and nerylacetone (**35**) was obtained from linalyl acetoacetate (**33**). Shimizu and Ishii reported a useful synthetic method of trifluoromethyl ketone **37** by the palladium-catalyzed reaction of allyl ***γ***, ***γ***, ***γ***-trifluoroacetoacetate **36**.9)

**Figure:**
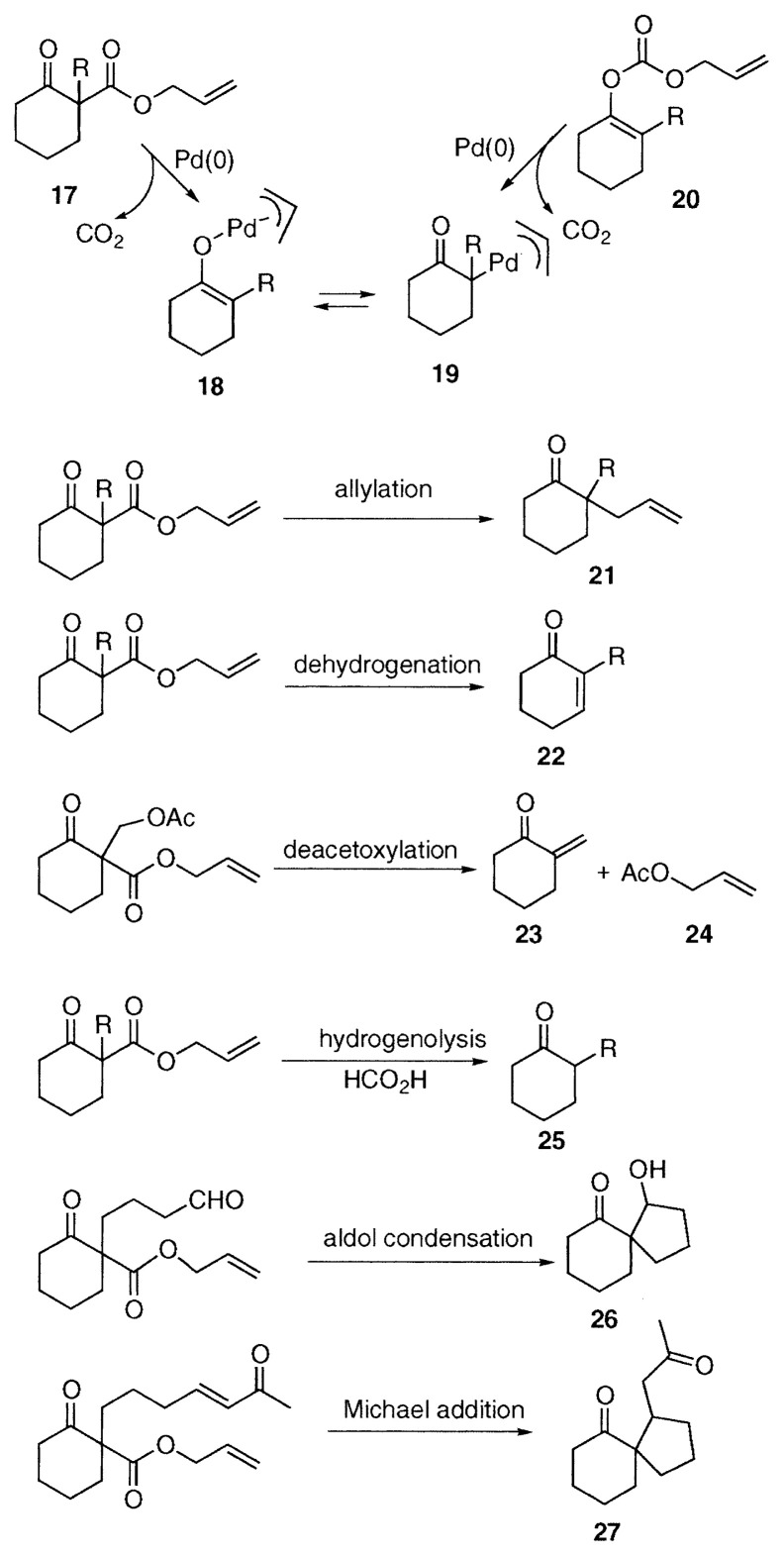


**Figure:**
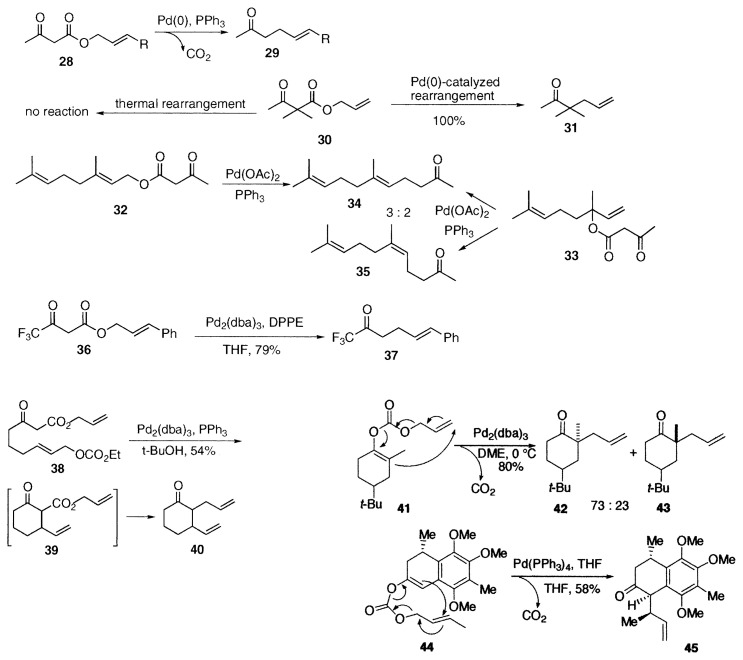


Allyl ***β***-keto carboxylate **38** having allyl carbonate moiety was converted to 2-allyl-3-vinylcyclohexanone (**40**). In this case, **39** was formed preferencially by intramolecular allylation of the ***β***-keto ester, and the subsequent Carroll rearrangement of **39** afforded **40**.10)

Several compounds related to allyl ***β***-keto esters undergo similar allylation reactions. Allyl enol carbonates **20** are typical examples. The enol carbonate **41** of 2-methyl-4-(*tert*-butyl)cyclohexanone underwent facile rearrangement at 0 ˚C in DME to afford a mixture of the stereoisomers **42** and **43** in 80% yield.8) As a recent application of regio- and stereoselective ***α***-allylation of cyclohexanone derivative, Nicolaou and coworkers carried out the palladium-catalyzed decarboxylation-allylation of the allyl enol carbonate **44** at room temperature to give the ***α***-allyl ketone **45** in 58% yield and a regioisomer (24%) using triphenylphosphine as a ligand in their total synthesis of colomviasin A.11) Paquette and coworkers applied a similar reaction of allyl enol carbonate to synthetic approach toward a phorbol framework.12)

As a related reaction, the silyl enol ether **47** was prepared regioselectively from the silyl enol ethers **46** of allyl ***β***-keto carboxylate at room temperature using diphenylphosphinoethane (DPPE) as a ligand. The silyl enol ether **47**, prepared in this way, was allylated further to give diallyl ketone **48** by the palladium-catalyzed reaction with diallyl carbonate.13)

**Figure:**
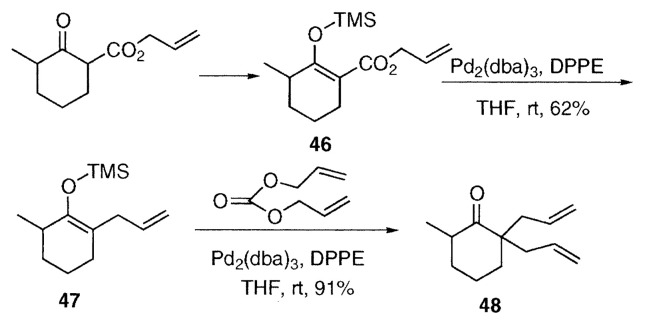


In addition to allyl ***β***-keto esters, derivatives of diallyl malonate **49** and allyl cyanoacetate **51** undergo similar decarboxylation-allylation to give allyl ***α***-allyl carboxylate **50** and ***α***-allylnitrile **52** accompanied by **53** respectively. The nitro ester **54** is very reactive and the allylation proceeds even at −50 ˚C to give the ***α***-allyl nitro alkane **55**.7)

**Figure:**
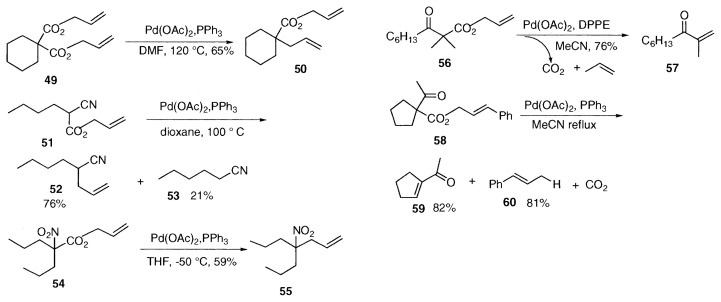


**Figure:**
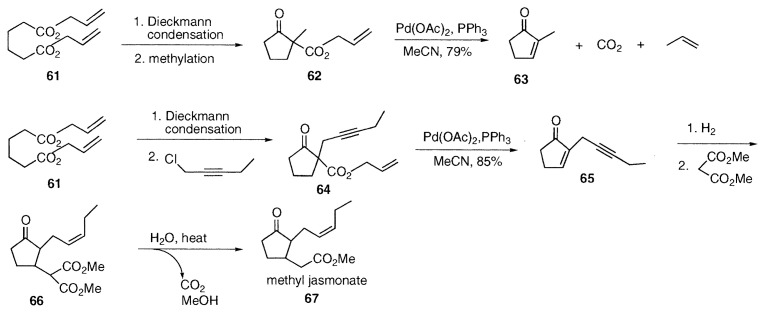


## Preparation of ***α***, ***β***-unsaturated carbonyl compounds by ***β***-hydrogen elimination

As the second reaction, we confirmed that elimination of ***β***-hydrogen from the palladium-enolates **18** or **19** affords ***α***, ***β***-unsaturated ketones **22** as expected in boiling acetonitrile. 14),15) ***α***, ***β***-Unsaturated ketone **57** was obtained from allyl ***α***, ***α***-dimethylacetoacetate derivative **56** in refluxing acetonitrile. The allyl group trapps the hydrogen and generates propene. As a supporting evidence, reaction of the cinnamyl ester **58** afforded the enone **59** and 1-phenylpropene (**60**) in equal amounts.

2-Substituted cyclopentenones are conveniently prepared by Dieckmann condensation of diallyl adipate (**61**), and alkylation, followed by palladium-catalyzed decarboxylation-dehydrogenation. Facile preparation of 2-methylcyclopentenone (**63**) from **62** is an example. 16) A commercial process for methyl *cis*-jasmonate (**67**) was developed applying the enone formation as a key reaction. Preparation of ***α***-(2-pentynyl)cyclopentenone (**65**) in 85% yield from allyl ***α***-(2-pentynyl)cyclopentanonecarboxylate (**64**) was carried out by the palladium-catalyzed ***β***-hydrogen elimination in boiling acetonitrile. Methyl *cis*-jasmonate (**67**) was obtained by hydrogenation of the triple bond in **65** to *cis* double bond, and Michael addition of dimethyl malonate to form **66**, followed by decarboxylation.17)

The enones are also prepared from enol carbonates **20**. 2-Methyl-2-cyclohexenone (**69**) was prepared regioselectively from the enol carbonate **68** of 2-methylcyclohexanone.18) The ***α***, ***β***-unsaturated aldehyde **72** was obtained from the enol carbonate **71** of aldehyde **70**.15) Similarly ***α***, ***β***-unsaturated nitrile **74** was prepared from the disubstituted allyl cyanoacetate **73**.15),19)

## Synthesis of ***α***-methylene carbonyl compounds by deacetoxylation

Acetoxymethyl group is introduced at ***α***-carbon of allyl cyclopentanonecarboxylate by the reaction of formaldehyde, followed by acetylation to give **75**. Unexpectedly the ester **75** underwent facile palladium-catalyzed decarboxylation and deacetoxylation to give ***α***-methylenecyclopentanone **77** and **23**.19),20) Interestingly, the acetoxy group in **76** was eliminated selectively as allyl acetate (**24**) more easily than ***β***-hydrogen, and the cyclopentenone **78** was not formed. Diallyl malonates undergo similar transformation. As a synthetic example, itaconate **82** was prepared from diallyl malonate. The appropriately substituted diallyl malonate **80** was prepared from diallyl malonate **79**, and subjected to the palladium-catalyzed reaction at 40 ˚C to provide allyl ethyl itaconate (**82**) as shown by **81** in 83% yield.

**Figure:**
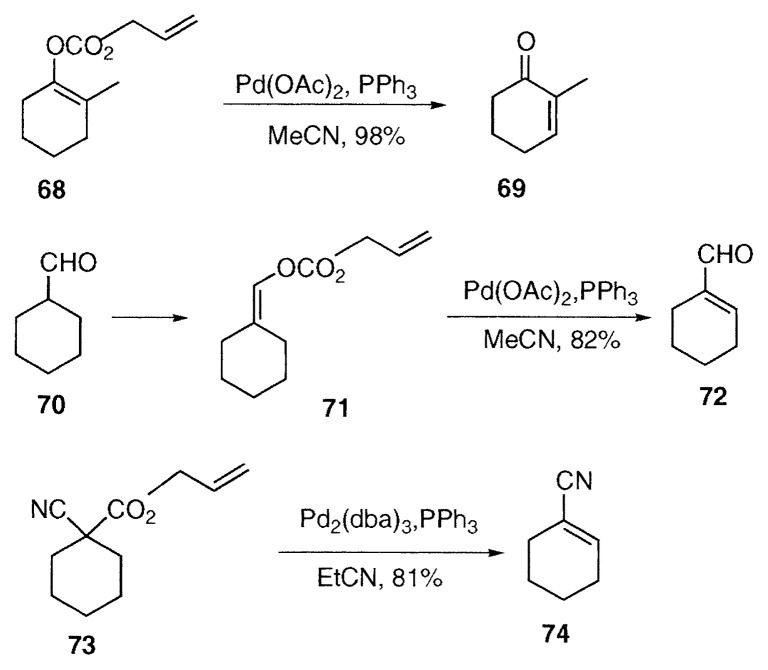


The reaction can be extended to other allyl acetates bearing EWG. The ***α***-methylenelactone **84** was prepared from allyl lactonecarboxylate **83** in acetonitrile at 50 ˚C. Nitro and sulfonyl groups are more reactive, and the reactions of **85** and **87** proceeded smoothly at 25 ˚C to afford 1-alkyl-1-nitroethylene **86** and 1-alkyl-1-sulfonylethylene **88** in good yields. Lactams are less reactive, and ***α***-methylene-***γ***-lactam **90** was prepared at somewhat higher temperature (80 ˚C) from the allyl ***γ***-lactamcarboxylate **89**.21) ***α***-Methylenelactones and cyclic ***α***-methyleneketones are present in natural products and show interesting biological properties, but the preparation of these labile functional groups in high yields under mild conditions is not easy. The palladium-catalyzed reaction which can be carried out under mild neutral conditions offers a useful synthetic method of these compounds.

**Figure:**
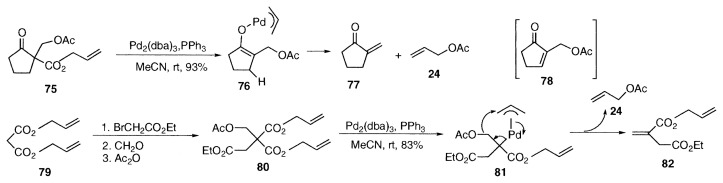


## Removal of the ester groups from substituted ***β***-keto esters and malonates by hydrogenolysis

In the classical uses of ***β***-keto esters and malonates, alkylated ***β***-keto esters and malonates are hydrolyzed and decarboxylated to afford alkylated ketones and esters, or acids. But the hydrolysis of congested disubstituted ***β***-keto esters and malonates is not easy, and usually harsh conditions are required. Sometimes rather strong bases or acids are used for hydrolysis. In addition, decarboxylation occurs at high temperature. On the other hand, palladium-catalyzed decarboxylation-hydrogenolysis of substituted allyl ***β***-keto esters and malonates proceeds at room temperature under neutral conditions using a mixture of formic acid and triethylamine, which is soluble in organic solvents, to give **25**. The reaction offers a useful method particularly for labile compounds.22) The reaction can be understood by the following mechanism. The palladium enolate **92**, formed from **91** is attacked by a proton from formic acid to afford the ketone **93** and ***π***-allylpalladium formate **94**. Decarboxylation of **94** generates ***π***-allylpalladium hydride **95** which collapses to propene and Pd(0).

Acid-sensitive esters such as THP-protected allyl ***β***-keto ester **96** was converted to **97** at room temperature without deprotection of THP group, and the dike-tone **99** was obtained from a base-sensitive ester **98** without undergoing retro-Michael addition.22) The method was utilized by Murai and coworkers in the synthesis of the glycinoeclepin intermediate **101**. Only the allyl ester in **100** was cleaved selectively to give the ketone, which was converted to trflate **101** without attacking the methyl ester and acetate in **100**.23)

Shimizu and coworkers reported an interesting synthetic method of ***α***-keto carboxylic acids such as **105**. The diallyl 2-oxosuccinate **104** was prepared from diallyl oxalate (**102**) and allyl 3-phenylpropionate (**103**), and chemoselective decarboxylation of only the allyl ***β***-keto carboxylate in **104** took place to give the ***α***-keto carboxylic acid **105**.24) Also Shimizu and Ishii obtained ***α***-fluorocyclododecanone (**106**) by hydrogenolysis of allyl ***α***-fluoro-***β***-cyclododecanonecarboxylate (**107**). In addition, they prepared ***α***-fluorocyclododecenone (**108**) from **107**.25)

Disubstituted diallyl malonates react smoothly with a mixture of formic acid and triethylamine. The free monocarboxylic acid **110** was obtained smoothly in a good yield in boiling dioxane from the disubstituted diallyl malonate **109**.26) As an application, reaction of the diallyl malonate attached to ***β***-lactam **111** proceeded stereoselectively to give the monocarboxylic acid with the ***β***-oriented methyl group **112**.27)

**Figure:**
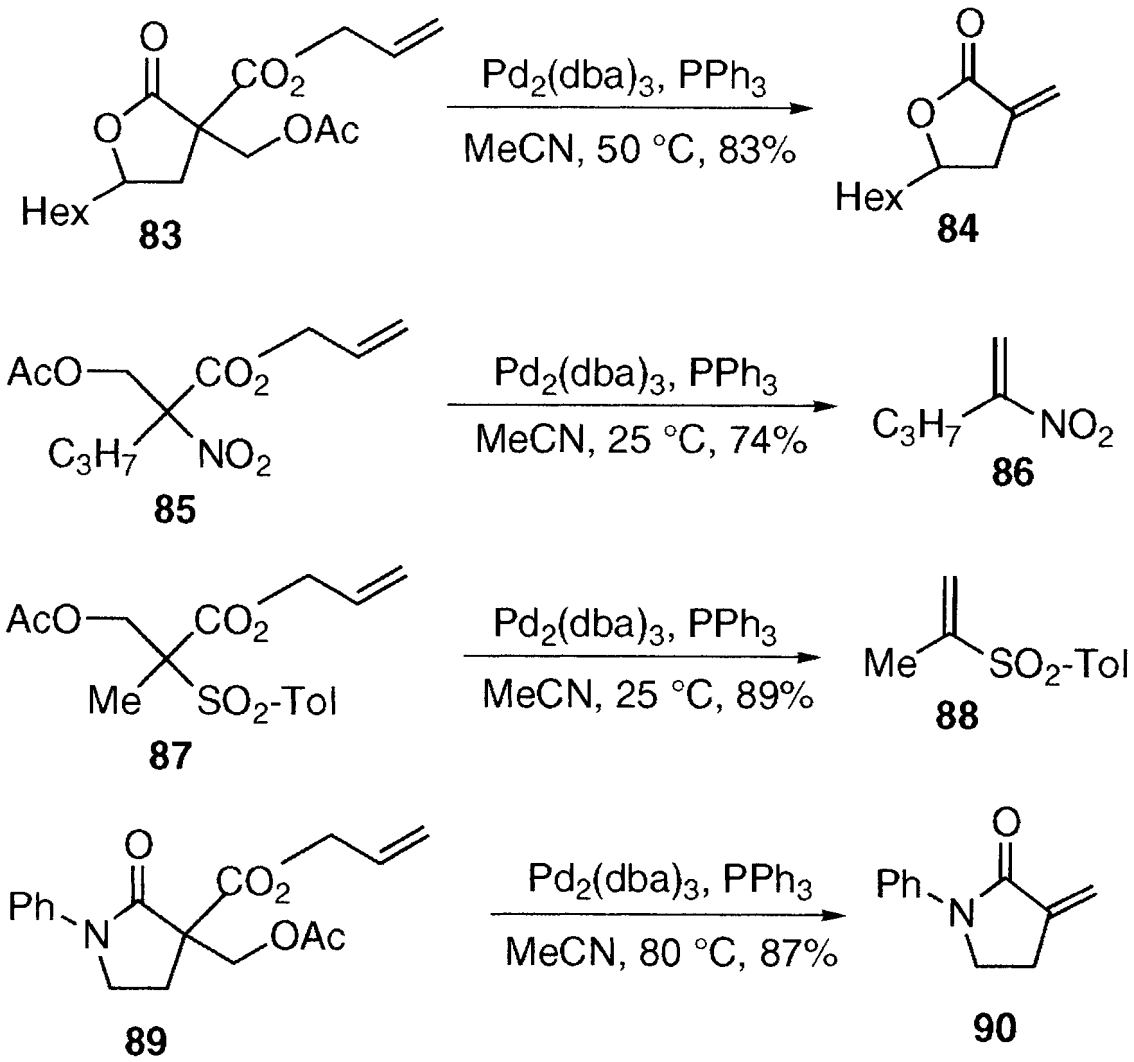


***γ***-Methylene-***γ***-butyrolactone **116** was prepared in one-pot in 81% yield from diallyl alkyl(2-propynyl)malonate **113** by palladium-catalyzed cyclization and hydrogenolysis with formic acid.28) In this case, after oxidative addition, intramolecular oxypalladation of the triple bond in the intermediate **114** takes place to afford the (***π***-allylpalladium)alkenyllactone **115**. Finally hydrogenolysis of **115** with formic acid provides ***γ***-methylene-***γ***-butyrolactone **116**.

## Palladium-catalyzed aldol condensation

Typical reactions of metal enolates are aldol condensation and Michael addition. As expected, we found that palladium enolates undergo these reactions smoothly. So far intramolecular reactions proceed efficiently, and intermolecular reactions are competitive with other reactions and hence selectivity is low. Palladium-catalyzed aldol condensation of allyl acetoacetate derivative **117** at room temperature gave the aldol **118** in 82% yield. When an aldehyde group is present in allyl ***β***-keto ester **119**, intramolecular aldol condensation took place yielding the cyclic aldol **122** in 96% yield and the diketone **122** as a minor product. In this reaction, the palladium alkoxide **121** is generated at room temperature *via* palladium enolate **120** and provides the aldol product **122**. No allylation occurs.29) The reaction of the allyl cyanoacetate **124** provided the lactone **126** in 56% yield as a main product and the alcohol **127** in 16% by intramolecular reaction of the intermediate **125**, followed by lactonization. The aldol condensation proceeds under neutral conditions.

**Figure:**
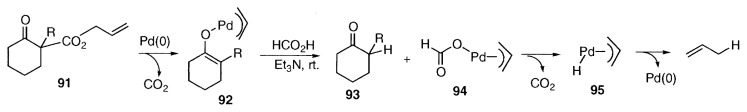


## Palladium-catalyzed Michael addition

Intra-molecular Michael addition *via* palladium enolates occurs under mild conditions.30) Michael addition of the allyl ***β***-keto ester **128** proceeded as shown by **129** to provide the cyclized palladium enolate **130** as an intermediate, and the enolate **130** was converted to the diketone **131** in 77% yield by protonation together with the cyclopentanones **132** and **133** as minor products formed by reductive elimination and ***β***-hydrogen elimination. Intramolecular Michael addition of the substituted diallyl malonate **134** occurred as shown by **135** to afford the cyclized product **136** as a main product and **137** as a minor one. Yamamoto and coworkers obtained the ketone **140** by the reaction of allyl acetoacetate (**138**) with an activated olefin **139**. The reaction is explained by Michael addition of the enolate **141** to **139** to give **142**, followed by allylation.31)

**Figure:**
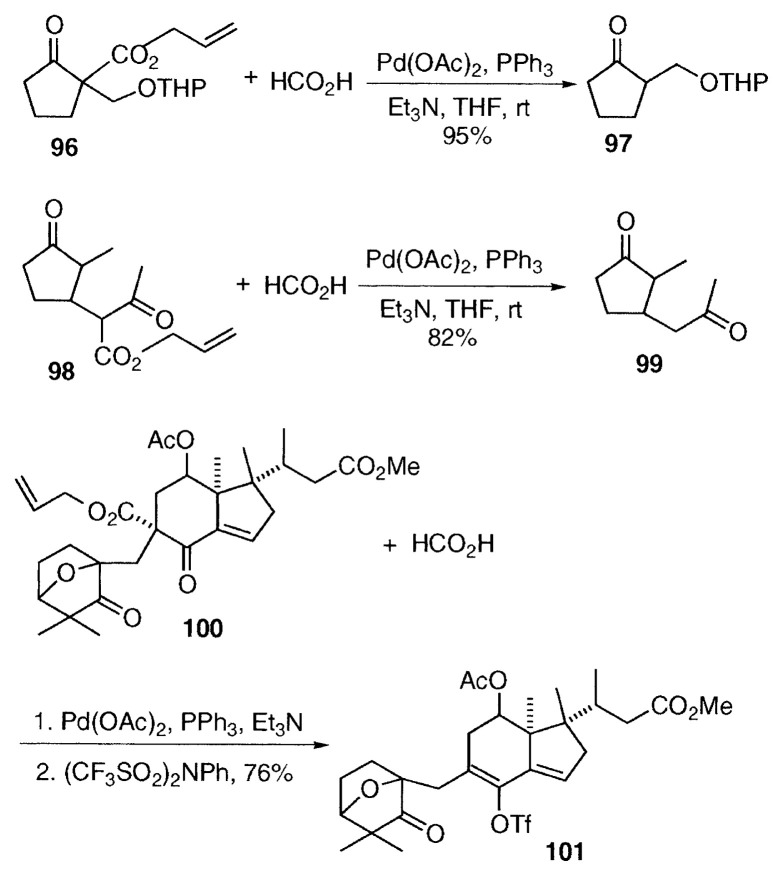


An interesting strategy for convergent steroid synthesis has been reported by Deslongchamps based on palladium-catalyzed domino decarboxylation-Michael addition of allyl ***β***-keto ester (bicyclic Nazarov reagent) **143** to the cyclohexenone **145**. The intermolecular Michael addition of the first palladium enolate **144**, generated from **143**, to **145** affords the second palladium enolate **146**. Intramolecular Michael addition of **146** provides the third enolate **147**, constructing finally the tetracyclic steroid skeleton **148** by ***β***-hydrogen elimination. 32)

**Figure:**
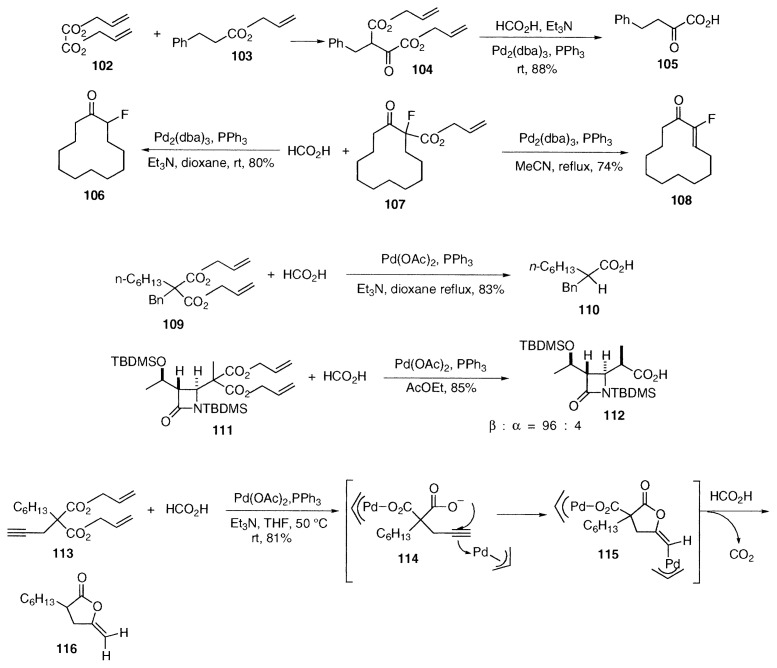


## Conclusions

Palladium enolates are generated from allyl ***β***-keto carboxylates after facile decarboxylation by the treatment of with a zero-valent palladium complex as a catalyst, and undergo several transformations. Reductive elimination provides ***α***-allyl ketones, and ***α***, ***β***-unsaturated ketones are obtained by elimination of ***β***-hydrogen. A new synthetic method for ***α***-methylene ketones was discovered. The allyl ester group can be removed easily by the treatment with formic acid and triethylamine to give ketones. Intramolecular aldol condensation and Michael addition proceed under neutral conditions. In addition to allyl ***β***-keto carboxylates, allyl acetates bearing electron-withdrawing groups such as malonates, cyanoacetate, and nitroacetates undergo similar transformations. Developments of these reactions expanded the classical chemistry of ***β***-keto carboxylates and malonates in a large extent.

**Figure:**
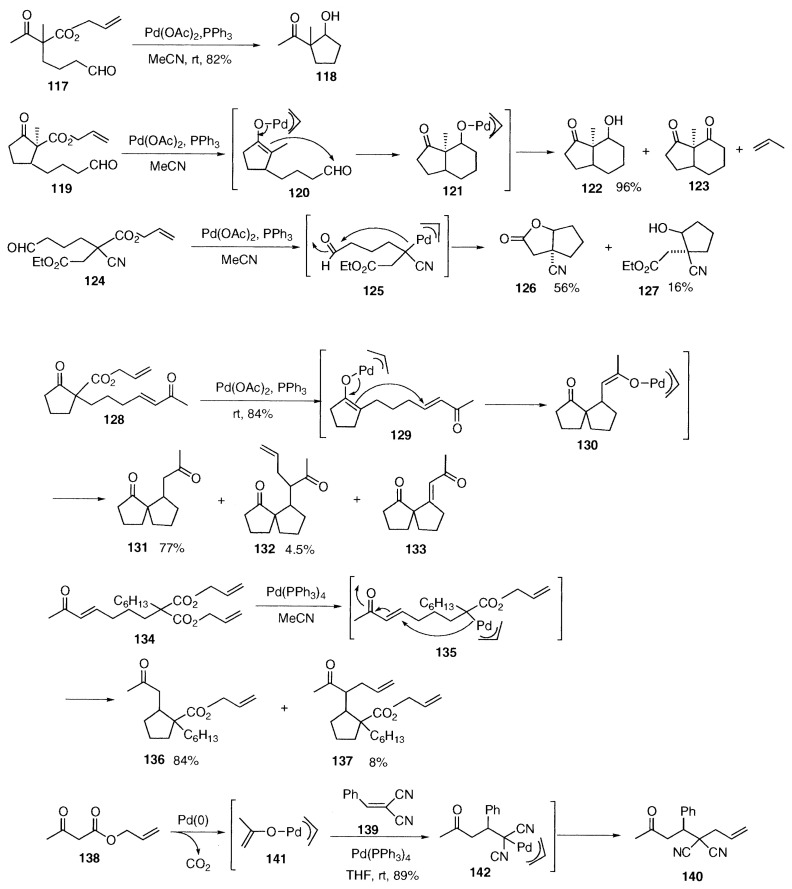


**Figure:**